# Supplementary material for: MAxSIM: multi-angle-crossing structured illumination microscopy with height-controlled mirror for 3D topological mapping of live cells
Source: Commun Biol. 2023 Oct 12;6:1034. doi: 10.1038/s42003-023-05380-2 (PMC10570291; doi:10.1038/s42003-023-05380-2)
Supplement: Supplementary file 2 — Supplementary Information [file 42003_2023_5380_MOESM2_ESM.pdf]

MAxSIM: Multi-Angle-Crossing Structured Illumination Microscopy with Height-Controlled Mirror for 3D Topological Mapping of Live Cells

Pedro Felipe Gardeazabal Rodriguez[1], Yigal Lilach[2], Abhijit Ambegaonkar[3], Teresa Vitali[1], Haani Jafri[4], Hae Won Sohn[3], Matthew Dalva[4,†], Susan Pierce[3], Inhee Chung[1,5*]

1 Department of Anatomy and Cell Biology, George Washington University, School of Medicine and Health Sciences, Washington, DC, USA
2 Nanofabrication and Imaging Center, George Washington University, Washington, DC, USA
3 Laboratory of Immunogenetics, National Institute of Allergy and Infectious Disease, National Institutes of Health, Rockville, MD, USA
4 Department of Neuroscience, Thomas Jefferson University, Philadelphia, PA, USA
5 Department of Biomedical Engineering, GW School of Engineering and Applied Science, George Washington University, Washington, DC, USA

† Current address: Department of Cell and Molecular Biology, Tulane University, New Orleans, LA 70118

* Correspondence to: inheec@gwu.edu

SUPPLEMENTARY INFORMATION

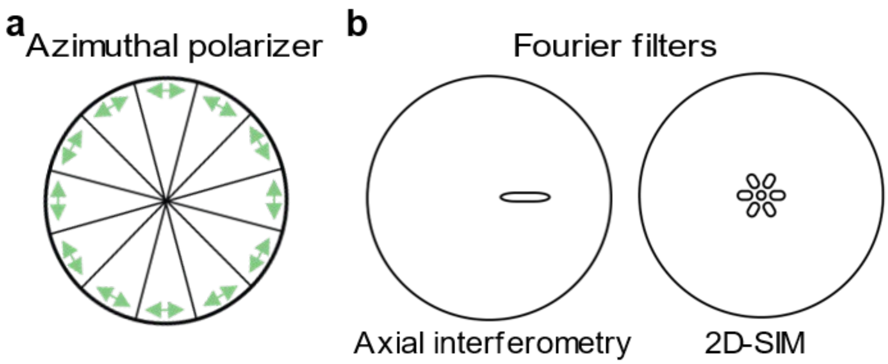

Supplementary Fig. 1. a. Schematic of the azimuthal linear polarizer. **b.** Static Fourier filters for axial interferometry (left) and 2D-SIM (right). The +1st and/or -1st diffraction orders of the s-polarized beams are selected by the filter after passing through the transposing polarizer segments.

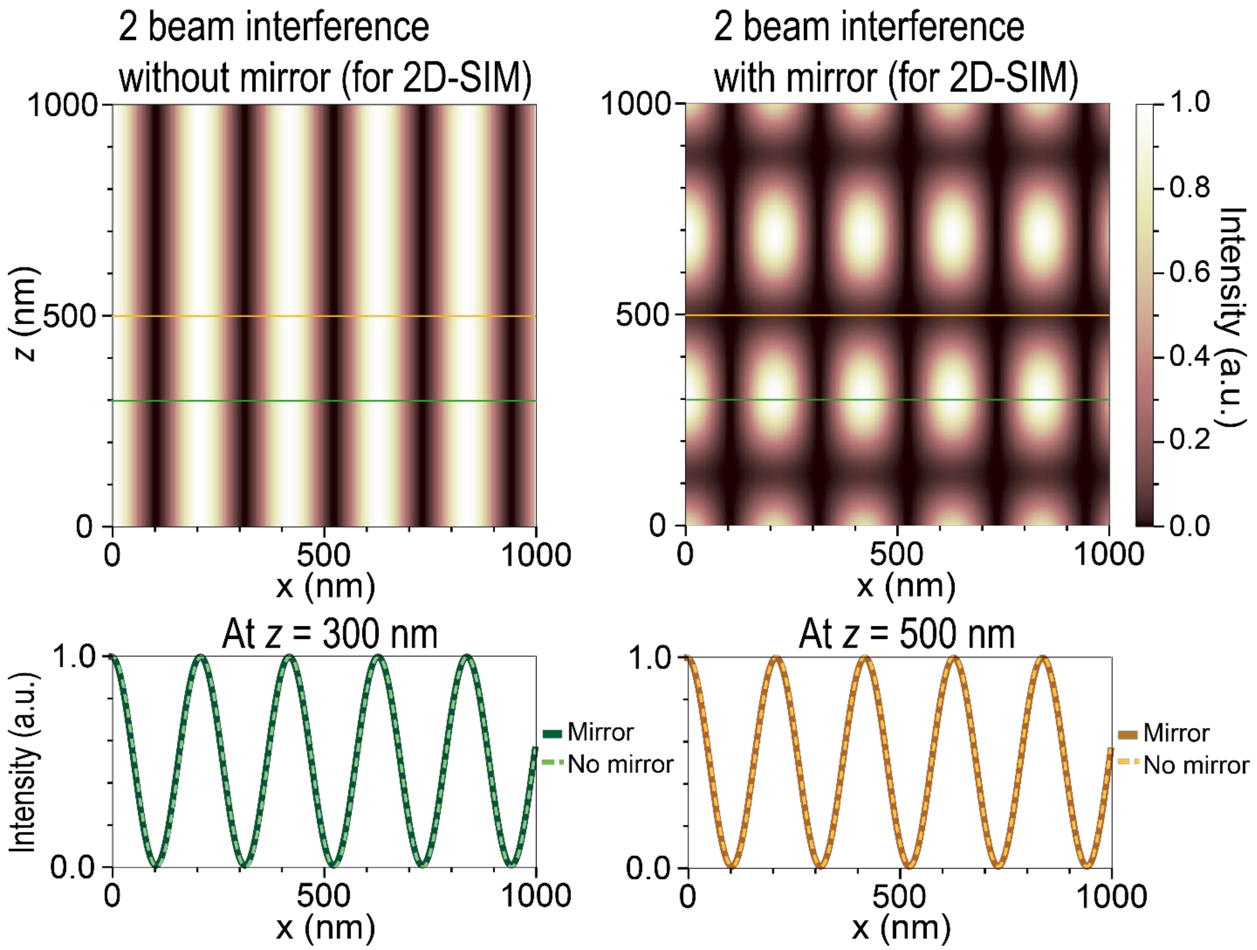

Supplementary Fig. 2. Top: Simulated results of two-beam (±1st-order beams) interference pattern in *x*-*z* without (left) and with (right) a mirror located perpendicular to the optical axis. The following constant values were used for the simulation: λ = 488 nm, θ = 60°, n_{si} = 4.37, n_{oxide} = 1.46, n_{media} = 1.33, and oxide layer thickness = 1,000 nm. Bottom: Lateral interference profiles at two *z* positions (300 nm, green; 500 nm, orange) show that lateral interference pattern in the 2D-SIM geometry without a mirror (left; dashed line) remains intact when a mirror is positioned perpendicular to the optical axis (right; solid line). Dashed lines and solid lines perfectly overlap.

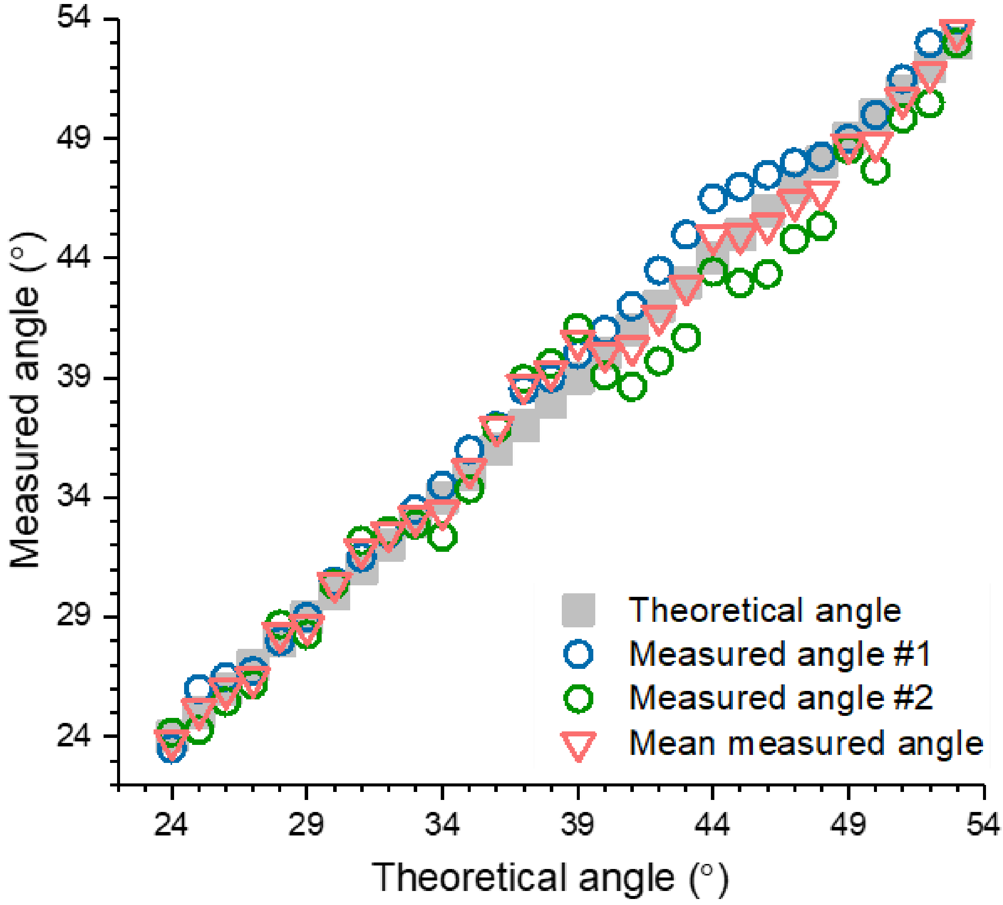

Supplementary Fig. 3. The calibration curve compares the measured incidence angles (two independently measured angles: blue and green circles; mean values: red triangles) produced by the SLM (Measured angle; Y-axis) with the theoretical angles used to create the input grating patterns for the SLM (Theoretical angle; X-axis). Supplementary Data 1 demonstrates two independent measurements (n=2) taken to provide mean and standard deviation values. The plotted values indicate that the measured incidence angles are < 2% of the theoretical angles.

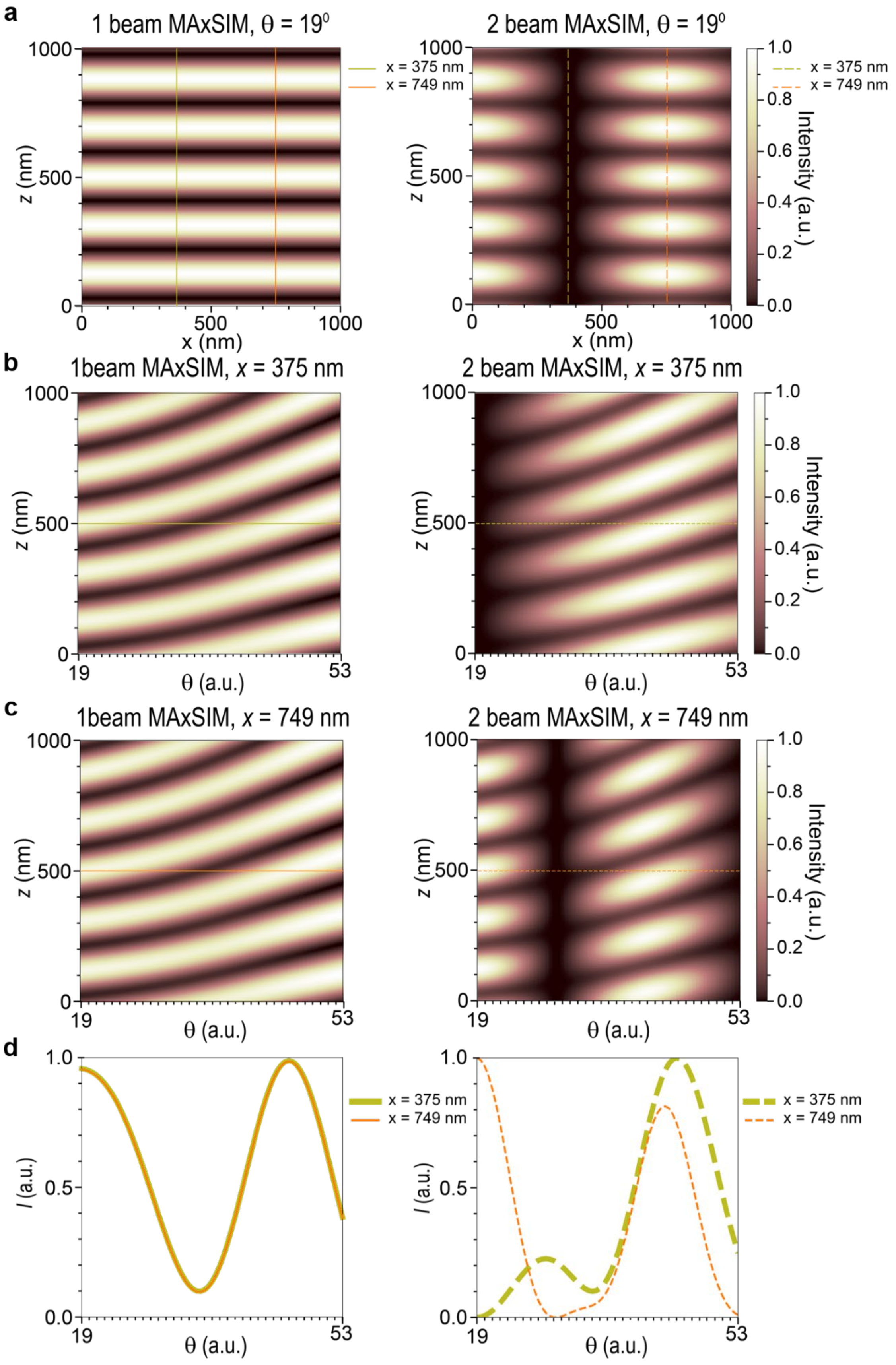
a
1 beam MAxSIM, θ = 19⁰
2 beam MAxSIM, θ = 19⁰
x = 375 nm
x = 749 nm
Intensity (a.u.)
z (nm)
x (nm)
b
1beam MAxSIM, x = 375 nm
2 beam MAxSIM, x = 375 nm
θ (a.u.)
c
1beam MAxSIM, x = 749 nm
2 beam MAxSIM, x = 749 nm
d
I (a.u.)

Supplementary Fig. 4. a. Simulated results of one- and two-beam (±1st-order beams) interference patterns in *x*-*z* at an incidence angle θ = 19° with the presence of a mirror located perpendicular to the optical axis. The two vertical lines (left, solid; right, dashed) are located at minimum (*x* = 375) and maximum (*x* = 749) intensity locations shown in the two-beam case. The following constant values were used for all shown simulation curves: λ = 488 nm, n_{si} = 4.37, n_{oxide} = 1.46, n_{media} = 1.33, and oxide layer thickness = 1,000 nm. **b,c**. Intensity profiles in θ-*z* dimensions at the two *x* positions defined in (a), as θ° varies from 19° to 53° with a step of 0.5° for one- and two-beam cases. Orange (left, solid) and green (right, dashed) lines indicate z = 500 nm. **d**. Intensity modulation profiles along the lines shown in (b,c) are overlaid for one-beam (left, solid) and two-beam (right, dashed) cases, showing that modulation patterns vary laterally for the two-beam case but are laterally invariant for the one-beam case. Since it is more straightforward to analyze laterally constant axial interference patterns, we chose to use one-beam-based axial interferometry for MAxSIM.

Supplementary Fig. 5. Theoretical expansion of the optical theory for MAxSIM[1-3].

Assume a beam path of coherent *s*-polarized incident light interfering with its own reflection from the Si layer at position *p*, a distance *h* away from the SiO_2 layer of d_{ox} thickness, where $\vec{k}_{i(r)}$, $\vec{E}_{i(r)}$, $\theta_{i(ox)}$ are the wave vector, electric field, and incident angle to the medium (to Si) of the incident (reflected) wave, n_m is the refractive index of the medium, and $n_{ox(si)}$ is the refractive index of the SiO_2 (Si) layer.

The electric fields of the incident and reflected beams in the medium are expressed as

$$\vec{E}_i = \vec{E}_0 e^{i(\vec{k}_i \cdot \vec{r})}$$
$$\vec{E}_r = r_{eff}^{TE} \vec{E}_0 e^{i(\vec{k}_i \cdot \vec{r})} \tag{1}$$

, where r_{eff}^{TE} is defined as the effective transverse electric (TE) Fresnel reflection coefficient corresponding to light interactions in the SiO_2-Si layers (we assume 100% reflection from SiO_2-Si interface). The *k*-vectors for the incident and reflected beams with wavelength λ in the medium of refractive index n_m are expressed as

$$\vec{k}_i = (k\sin(\theta_i);\ 0;\ -k\cos(\theta_i))$$

$$\vec{k}_r = (k\sin(\theta_i);\ 0;\ k\cos(\theta_i)), \tag{2}$$

where $k = \frac{2\pi n_m}{\lambda}$.

Using Equation 1, the total electric field at point *p* is

$$\vec{E}_t = \vec{E}_0 e^{i(\vec{k}_i \cdot \vec{r})} \left(1 + r_{eff}^{TE} e^{i(\vec{k}_r - \vec{k}_i)\cdot\vec{r}}\right) \tag{3}$$

From Equation 2,

$$\left(\vec{k}_r - \vec{k}_i\right) \cdot \vec{r} = \frac{4\pi n_m}{\lambda} h\ \cos(\theta_i) \equiv \Phi(h) \tag{4}$$

, where $\Phi(h)$ is the phase difference between the incident and reflected beams in the medium at *h* above SiO_2. The intensity at point *p*, which is the square of the amplitude of the electric field at *p*, is expressed as

$$I_1 = \left|1 + r_{eff}^{TE} e^{i\Phi(h)}\right|^2 \tag{5}$$

The general expressions for the TE Fresnel reflection (at the interface between media 1 and 2) (*r*) and transmission (between media 1 and 2) (*t*) coefficients are

$$r = \frac{n_1\cos(\theta_i) - n_2\cos(\theta_t)}{n_1\cos(\theta_i) + n_2\cos(\theta_t)}$$
$$t = \frac{2n_1\cos(\theta_i)}{n_1\cos(\theta_i) + n_2\cos(\theta_t)} \tag{6}$$

, where θ_I and n_1 are the incidence angle and refraction index of the first medium, and θ_t and n_2 are the transmission angle and refraction index of the second medium. θ_t is derived from θ_I using Snell's law.

The incident beam that reflects off the Si layer is subject to continuous reflections within the SiO_2 layer at the SiO_2-Si and SiO_2-medium interfaces, creating a phase difference δ at each traversal within the SiO_2 layer. Thus, δ is defined as the product of the wave number times the traversed length by the beam in SiO_2, $2d_\mathrm{ox}\cos(\theta_\mathrm{ox})$. Thus,

$$\delta = \frac{4\pi n_\mathrm{ox}}{\lambda}\, d_\mathrm{ox}\cos(\theta_\mathrm{ox}). \tag{7}$$

The magnitude of the total electric field in the medium, A_total, becomes

$$A_{total} = \frac{r_\mathrm{m-ox}+r_\mathrm{ox-si}e^{i\delta}}{1+r_\mathrm{m-ox}r_\mathrm{ox-si}e^{i\delta}}A_0. \tag{8}$$

By writing the total amplitude in the medium as $A_\mathrm{total} = r_\mathrm{eff}^\mathrm{TE}A_0$, $r_\mathrm{eff}^\mathrm{TE}$ becomes

$$r_\mathrm{eff}^\mathrm{TE} = \frac{r_\mathrm{m-ox}+r_\mathrm{ox-si}e^{i\delta}}{1+r_\mathrm{m-ox}r_\mathrm{ox-si}e^{i\delta}}. \tag{9}$$

Supplementary Fig. 6. MAxSIM reconstruction algorithm.

Height reconstruction scheme for MAxSIM data

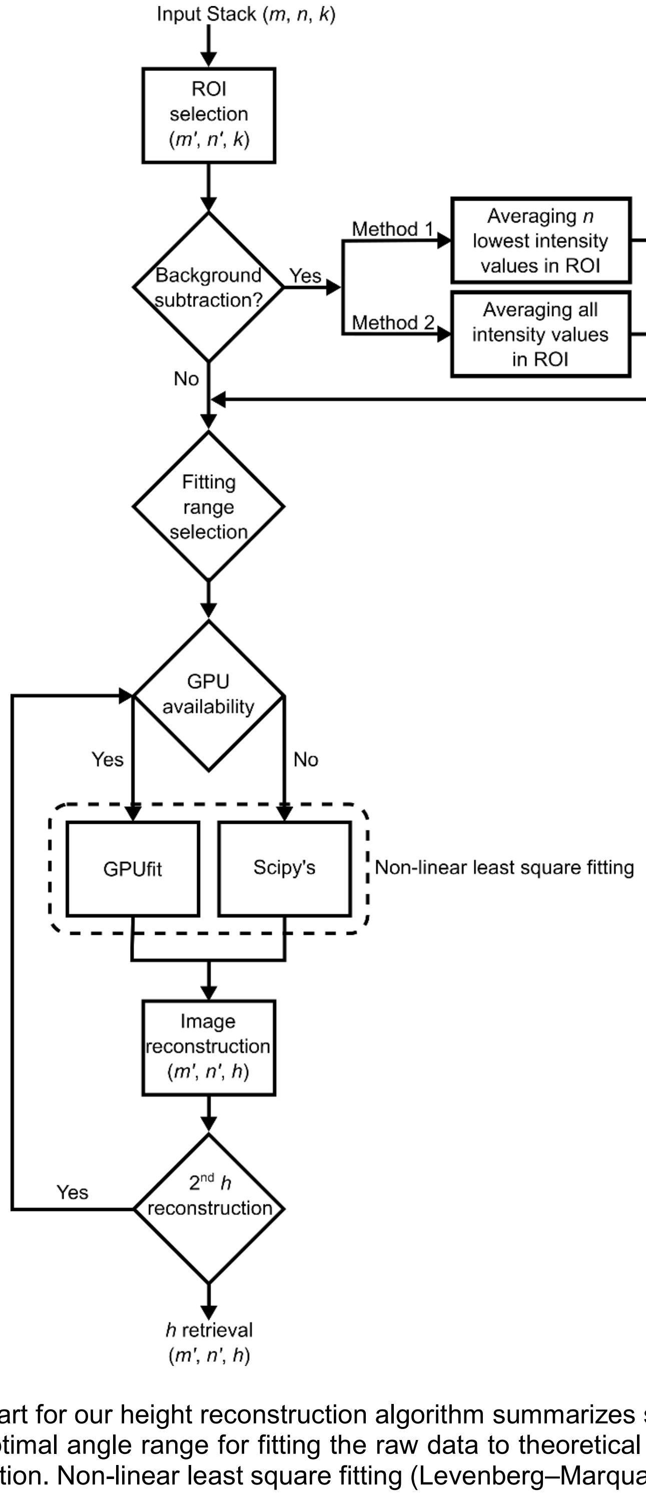

sub-figure S6a. Flowchart for our height reconstruction algorithm summarizes step-by-step actions in the Python environment. Optimal angle range for fitting the raw data to theoretical formula selected for high-fidelity height reconstruction. Non-linear least square fitting (Levenberg–Marquardt algorithm) was applied to the raw data at each pixel for height retrieval.

1. Input data

A raw MAxSIM dataset comprises a set of 2D images taken at *k* different incident angles θ_{air} within a range (sub-figure S6a). For instance, if the θ_{air} range is (19°, 53°) with a 0.5° step size as in our default setting, k is 68. The input stack dimension is thus (m, n, k), where m and n are the numbers of x and y pixels and k corresponds to the total number of θ_{air}.

* Our algorithm is optimized for fitting data with at least 4 interference fringes within the given angle range. However, it may not provide high-fidelity fitting for fluorescent objects placed on the SiO_2/Si mirrors unless SiO_2 >> 1 μm. In such cases, it is recommended to use the original Levenberg-Marquardt least square fitting algorithm instead.

2. ROI selection

Image reconstruction can proceed in a region of interest (ROI). An ROI can be defined as a rectangle or polygon. A user can define one rectangle ROI or multiple polygon ROIs at a given time. A left click on the mouse can be used to draw a polygon and a right click will complete one polygon.

3. Background subtraction

Background subtraction of the acquired 2D images can be crucial for high-fidelity least square fitting of the angle-dependent fluorescence intensity $I(\theta_{\mathrm{air}})$ data to the theoretical formula at a given pixel point. We offer two options to set the background intensity value in each image, by calculating the average value of: 1) the *n* lowest (user-defined) intensity values of the whole image, or 2) total intensity values within an ROI.

4. Customization of the Levenberg–Marquardt algorithm for *h* retrieval

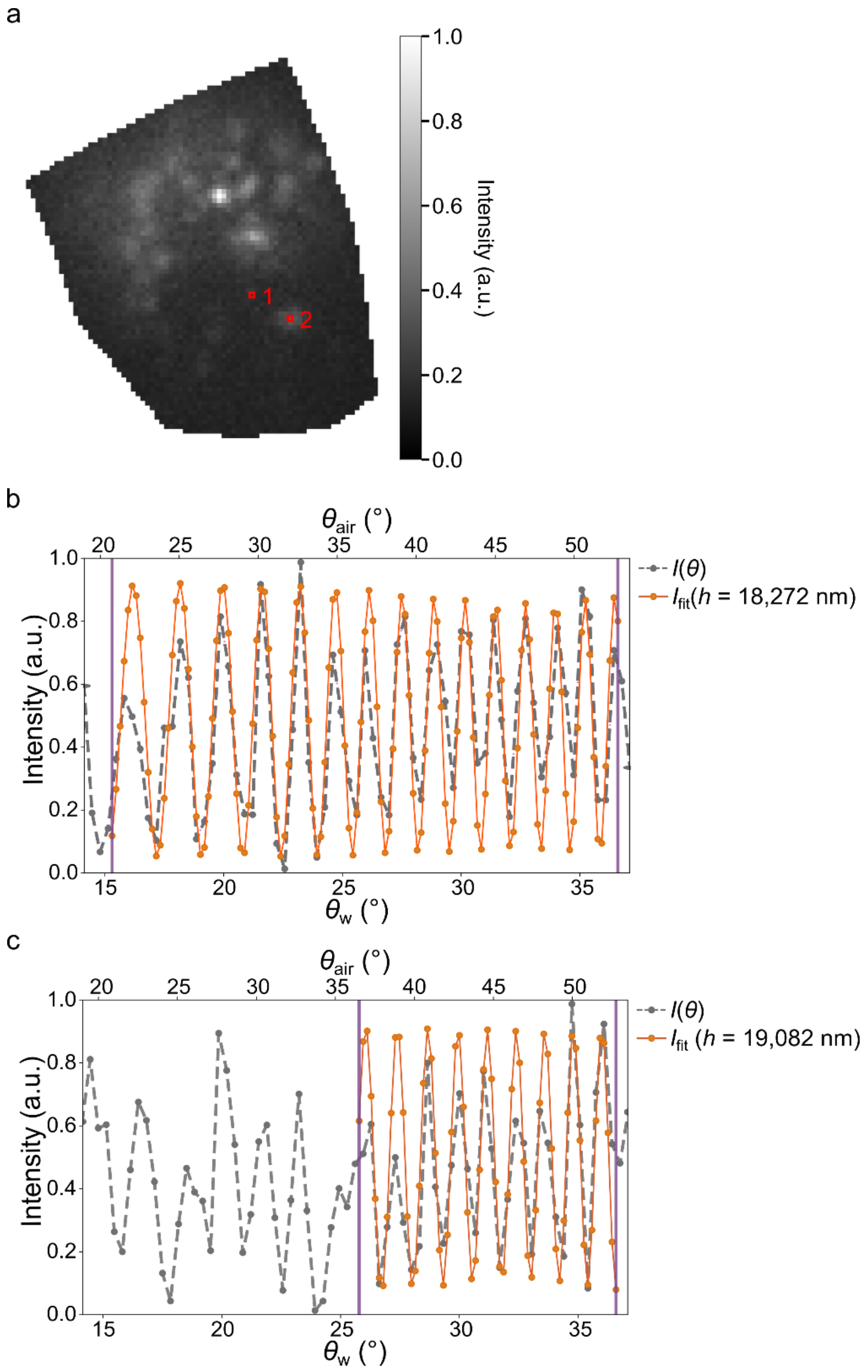

sub-figure S6b. a. A raw image of a fixed germinal center (GC) B cell taken at an incidence angle θ_{air} = 19° with excitation at λ = 560 nm. b-c. Raw ($I(\theta)$) and fitted (I_{fit}) plots at the two indicated pixel points in the image shown in (a). Normalized raw intensity data points (gray dots) to (0, 1) measured at each incidence angle (θ_{air}, top; θ_w, bottom) in the range of (19°, 53°) with 0.5° step size from pixel 1 (b) and 2 (c) in (a). Gray dotted lines connect the gray dots (measured intensity points). Orange line plot (I_{fit}) within the selected angle range (between two purple vertical bars) that was determined by our algorithm to give rise to the best fit (lowest NELD values) to the theoretical formula. Fitted orange curves (I_{fit}) are overlaid with $I(\theta)$.

We describe the height reconstruction procedure using the GC B cell example image shown in sub-figure S6b. sub-figure S6b-a is one MAxSIM raw image taken at θ_{air}= 19°, where two-pixel points are indicated with red squares to showcase the reconstruction process. sub-figure S6b-b,c shows the raw data (incident angle dependent fluorescence intensity plot, $I(\theta)$; gray dots) overlaid with the final fitted curves (I_{fit}, orange lines) in the angle ranges that were determined by our reconstruction algorithm for high-fidelity non-linear least square fitting based on the procedures described below.

4A. Data normalization and data point addition

The raw data at each pixel is given in the form of the fluorescence intensity value at each incident angle θ within a range. The raw data at each pixel is normalized between 0 and 1 as follows:

$$I(\theta) = \frac{I(\theta) - \min(I(\theta))}{\max(I(\theta)) - \min(I(\theta))} \quad (1)$$

In our case, $I(\theta)$ is generally acquired for θ_{air} between 19° and 53° with 0.5° step size. After normalization, a mean intensity point is located at each mid-angle point between the two adjacent data points for better fitting. This results in increased data points located at every 0.25° between 19° and 53° as shown in $I'(\theta)$ in sub-figure S6b-a,b.

4B. Determine the peaks and valleys that can be used for fitting

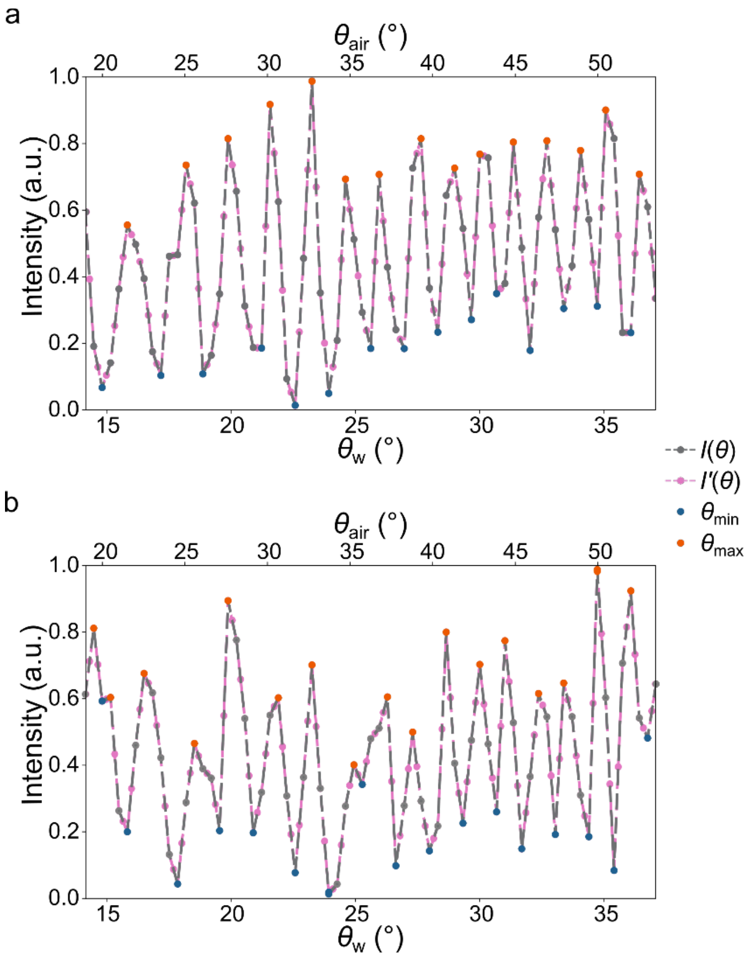

sub-figure S6c. Detection of the extrema locations of intensity modulation fringes. $I(\theta)$: normalized raw data points (gray dots) shown in sub-figure S6b-b,c that are connected by gray dashed lines; $I'(\theta)$: mean-intensity points (pale violet) between the two adjacent intensity values were added at the mid points of $I(\theta)$. Maxima (orange dots) and minima locations (teal dots) are identified.

We determine the locations of peaks (maxima points) and valleys (minima points) in $I'(\theta)$ shown in sub-figure S6c-a,b using Scipy's find_peak algorithm[4]. The following criteria are additionally used to further select the sub-angle range that yields the best fit by filtering out those peaks and valleys that do not meet the requirements below.

(a) The prominence, a parameter used in Scipy's algorithm that is the vertical distance of a peak (or a valley) from its maximum (minimum) to the extremum position in the subsequent valley (or a peak), should be greater than the standard deviation of y-values of $I'(\theta)$.

(b) The nearest neighbor's peak (valley)-to-valley (peak) distance should be greater than two *x* data points (in this case, 0.5°) (this constraint is used for the HCM ridge height >5 μm and 1 μm oxide layer).

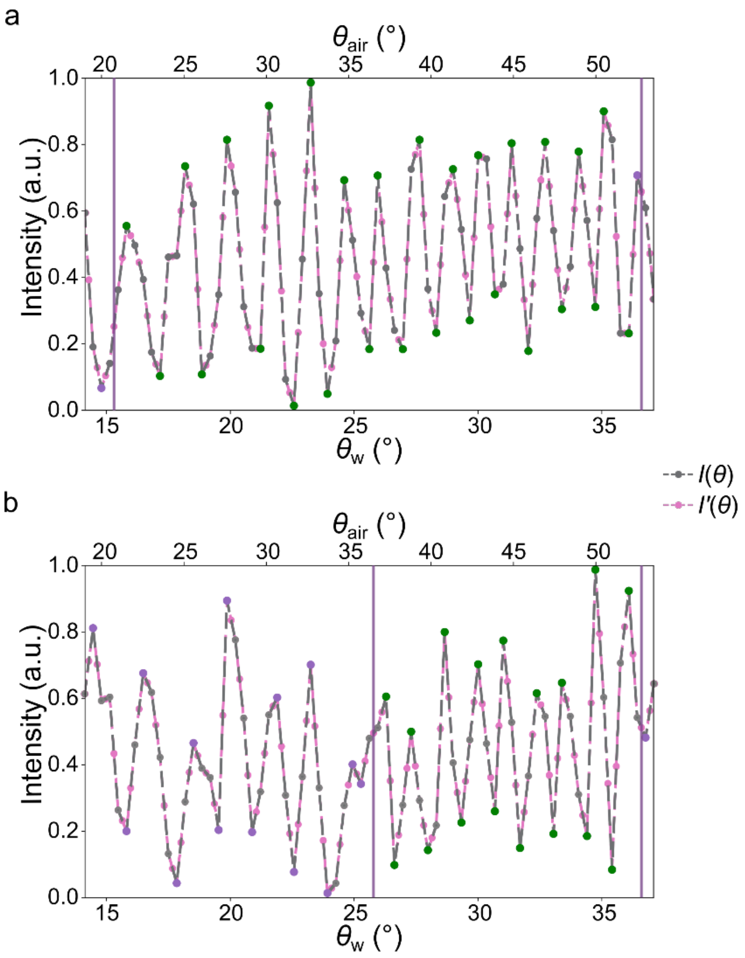

sub-figure S6d: Grouping peaks and valleys (extrema positions are indicated in green dots) that meet the selection criteria are applied to sub-figure S6c-a, b. Extrema positions that were filtered out are indicated as violet dots. Purple vertical lines show the angle range that contains the selected group in each processed plot.

Once those peaks and valleys are initially determined, we group those that contain more than three consecutive peak-valley pairs and apply consequent criteria to further narrow down to one group for fitting using the following criteria (sub-figure S6d).

(c) The y-axis distances between a maximum or minimum point to the subsequent minimum or maximum point must be greater than the threshold y-axis distance range, custom-defined using the average value of all maximum (or minimum)–minimum (or maximum) pairs in $I'(\theta)$. In addition, the x-axis distance between a maximum (or minimum) point to the successive minimum (or maximum) point must be within a specified custom-range based on the average spacing in $I'(\theta)$. To determine the range of the lower cut-off threshold for the y-axis and x-axis distance range that yields the best fitting, we use iteration. For the y-axis distance lower cut-off, we vary the lower cut-off values from 5%–55% of the average y-axis distance with a 5% increment to filter out peaks and valleys with smaller y-axis distances than the lower cut-off values by determining the cut-off value that minimizes the NELD value (see section 4C for description) using the Levenberg–

Marquardt non-linear least square fitting algorithm (see section 5). For x-axis distance range, we vary the distance range that can be created using any permutation of two numbers from 50%–100% of the averaged x-axis distance value with a 5% increment. Only those peaks and valleys that are within the x-axis distance range that was selected to minimize the NELD value can be grouped for further processing if more than two consecutive pairs can be found.

(d) To filter out peaks and valleys within groups whose y- and x-axis distances deviate significantly from the averaged values calculated from those within the groups, the selection process described in (c) is applied again to the data within the groups.

(e) Those remaining consecutive peaks and valleys are regrouped if there are more than two or three (user-defined) consecutive peak (or valley)–valley (or peak) pairs in a group.

We apply the Levenberg–Marquardt algorithm to fit the raw data within groups to the theoretical formula. After fitting is complete and *h* values are retrieved for the pixel, one selection criterion is applied to filter out those groups that did not meet the requirement.

(f) The total numbers of the extrema points between the sub-group plot of $I'(\theta)$ and the fitted plot I_{fit} must be identical.

If multiple sub-groups are found, the one with the minimum NELD value is chosen for height retrieval. If those sub-groups have the same NELD values, the one associated with the lowest NELD value for the entire angle range (not within the sub-group) is chosen. For a pixel that does not have any remaining sub-groups after applying the criteria above, *h* = -1 is assigned for the pixel, and these pixels are reprocessed during the second reconstructions.

4C. Metric for evaluating fitting uncertainty: normalized extrema location difference (NELD)

We created a metric called NELD to evaluate the fitting uncertainty by assessing the closeness between extrema positions between $I'(\theta)$ and I_{fit} as shown below.

$$\mathrm{NELD} = \frac{1}{n'+m'} \sum_{j=2}^{n+m-1} \frac{\sqrt{\left(\theta_{\mathrm{o}_j^{+(-)}} - \theta_{\mathrm{e}_j^{+(-)}}\right)^2}}{\left(\theta_{\mathrm{o}_{j+1}^{-(+)}} - \theta_{\mathrm{o}_{j-1}^{-(+)}}\right)} \tag{2}$$

, where *m* and *n* are the total numbers of maxima and minima, respectively, and *m*' and *n*' are the total numbers of peaks and valleys, respectively, in incident angle-dependent fluorescence intensity curves within an angle range. A peak of a valley is determined if a minimum or maximum location is situated between two adjacent maxima or minima. $\theta_{\mathrm{o}_j^{+}}$and $\theta_{\mathrm{o}_j^{-}}$ are the maximum (+) and minimum (-)-intensity angle locations, respectively, of the observed (o) and processed data plot $I'(\theta)$, while $\theta_{\mathrm{e}_j^{+}}$ and $\theta_{\mathrm{e}_j^{-}}$ are the maximum and minimum-intensity locations, respectively, of the expected (e) angles of the expected

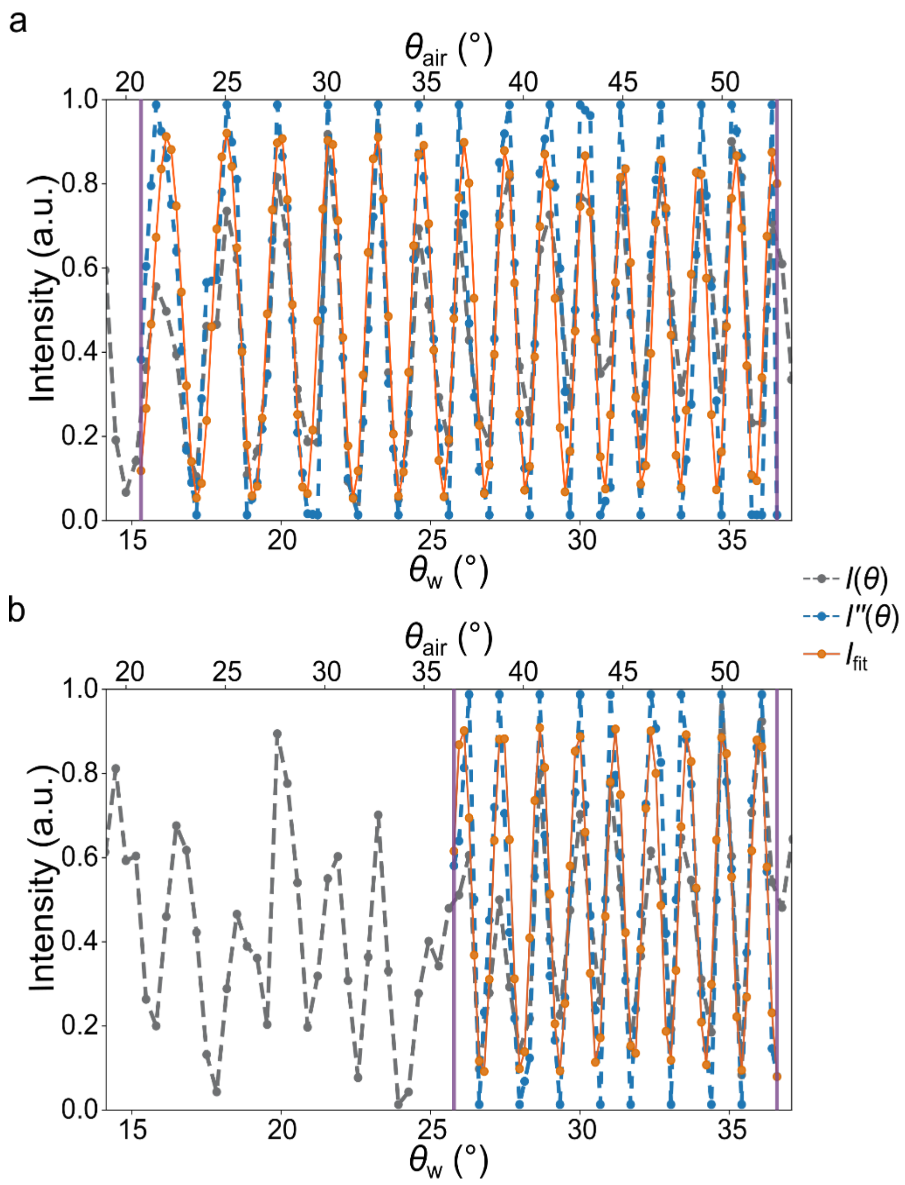

sub-figure S6e: a, **b**. Final fitted curves (I_{fit}, orange solid lines) overlay $I^{'}$ and I_{rs} , demonstrating the high-fidelity fitting capability of our reconstruction schemes.

(fitted) plot I_{fit}. Lastly, $\left(\theta_{\text{o}_{j+1}^{-}} - \theta_{\text{o}_{j-1}^{-}}\right)$ and $\left(\theta_{\text{o}_{j+1}^{+}} - \theta_{\text{o}_{j-1}^{+}}\right)$ are the sizes of an observed peak and valley, respectively. All angles are in radians.

5. Data repositioning within a selected sub-group

To improve the fitting fidelity, we first redefine the selected angle range by including three data points before the second appearing maximum or minimum points and after the second-last appearing maximum or minimum points. Then we adjust $I'(\theta)$ within our selected range, starting from three data points before the second maximum or minimum, whichever appears first. The ending angle is similarly determined using three data points after the second-to-last maximum or peak.

Then, we reposition all extrema positions to 1 (for maxima) or 0 (for minima), and the data points between them are re-scaled accordingly (as shown in sub-figure S6e).

6. Initialization and fitting

The theoretical formula describing the interference pattern of a beam interacting with its reflection from a mirror is denoted as:

$$I = \left|1 + r_{\mathrm{reff}} e^{i\phi}\right|^2 \tag{3}$$

or

$$I(\theta, h) = \left|1 + r_{\mathrm{eff}}(\theta) \mathrm{e}^{\mathrm{i}\phi(\theta, h)}\right|^2 \tag{4}$$

, where $r_{\mathrm{eff}}(\theta)$ is the effective reflection coefficient and ϕ is the phase shift between the incoming and outgoing beam, which depends on distance from the SiO_2/Si, h, that is retrieved from the Levenberg–Marquardt fitting. The observed fluorescence is proportional to the excitation intensity *I* (where *a* is a fitting constant) and noise (*b*) that is incorporated into the signal during image acquisition. So I_{fit} can be simplified as:

$$I_{\mathrm{fit}} = aI + b \tag{5}$$

or

$$I_{\mathrm{fit}} = aI(\theta, h) + b \tag{6}$$

The parameters to fit are then a, b, and h. Of the three, the parameter of interest is the distance (h) to SiO_2/Si. To obtain a high-fidelity fit, an excellent initialization of the parameters is critical due to the presence of local minima when optimizing the residual in a least-squares regression. Our processed raw data are contained between 0 and 1, so the amplitude is initialized to 1 and the bias to 0. However, for determining the height, we reference a lookup table that is created to detail the theoretical counts of maxima and minima based on the distance to SiO_2/Si (refer to the readme file associated with our

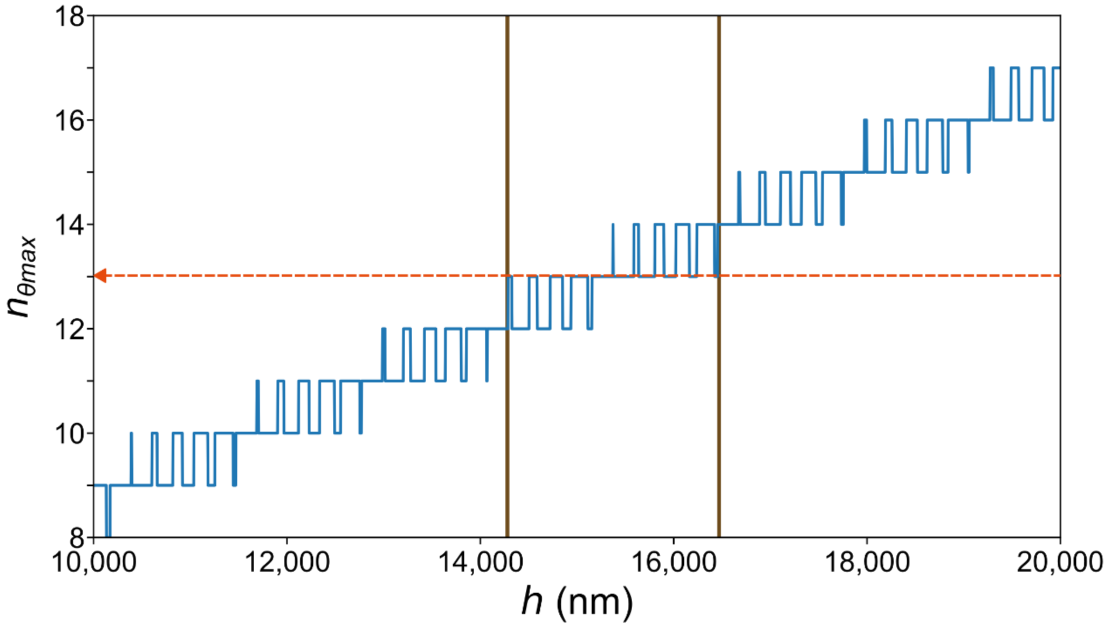

sub-figure S6f: The theoretical number of maxima (blue line) within the specified angle range as a function of *h.* If, for example, the raw data have 13 maxima points (indicated by the arrow of the red dashed horizontal line), the initial height parameters within the *h* range (between the two vertical bars) that give rise to the same maxima number will be tested to identify the one with the lowest NELD value.

reconstruction algorithm).

7. Height retrieval

For each initial height value tested for a single pixel fit, our algorithm logs the amplitude (a), bias (b), and height (h) from the Levenberg–Marquardt fitting, along with the NELD value for the corresponding angle range. The height corresponding to the minimal NELD value is chosen for that pixel (see sub-figure S6f). If there are multiple sets with identical minimal NELD values, we select the one linked to the minimum NELD value from the complete angle range.

8. Second *h* reconstruction

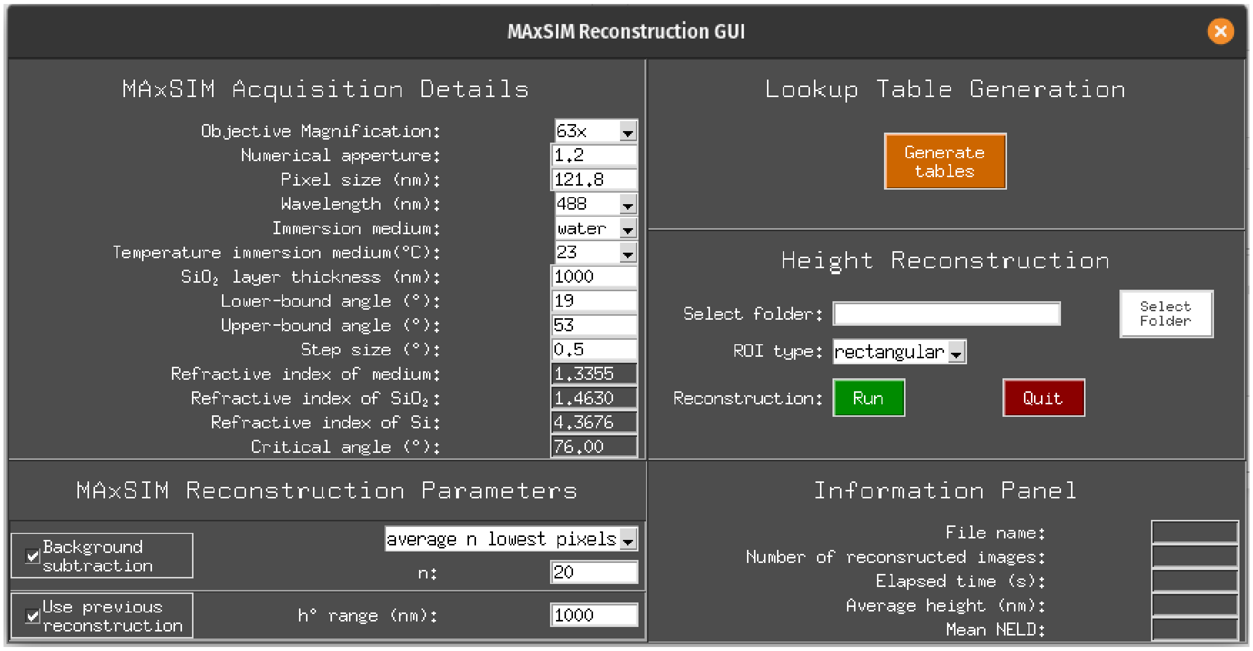

sub-figure S6g. Snaphot of the interface of our MAxSIM height reconstruction code.

A second height reconstruction is performed for those pixels with a height not retrieved ($h = -1$) from the first reconstruction. For the initial height parameter, we assign the h value of the nearest neighboring pixel with the lowest NELD value. Median filter can be applied to the height reconstructed data.

Additional details on the MAxSIM reconstruction algorithm interface, written in Python, are available in the Readme file. Sub-figure S6g provides a snapshot of our interface display.

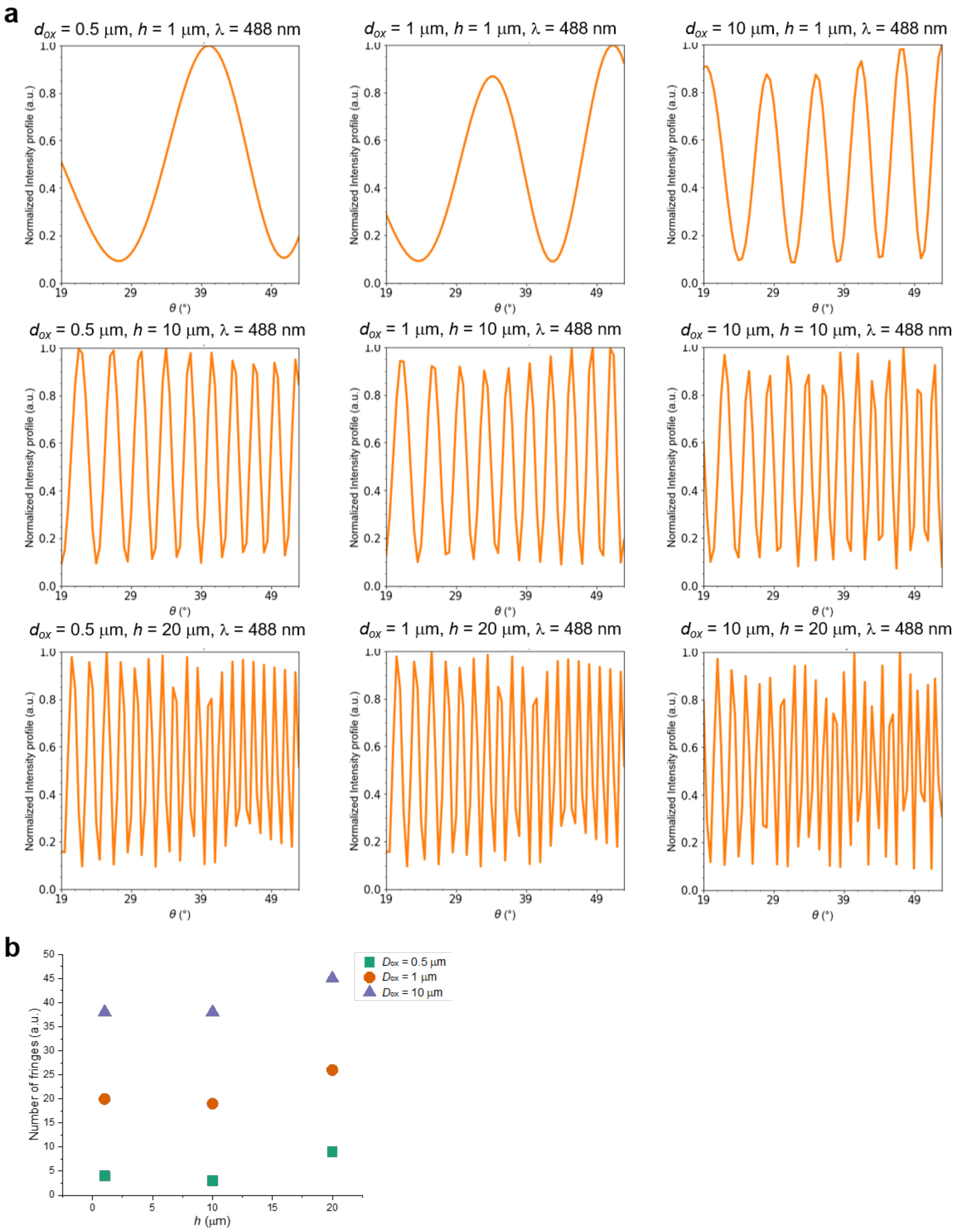

Supplementary Fig. 7. The excitation intensity interference pattern at λ = 488 nm was simulated for three different SiO_2 thickness cases: D_{ox} = 0.5, 1, and 10 μm, along with three different chromophore heights: h = 1, 10, and 20 μm from the SiO_2 layer. The results indicate that the number of interference fringes increases with larger values of D_{ox} and higher chromophore locations (h). **b.** The number of fringes was counted from the simulated curves (**a**) and plotted, revealing that the cases with D_{ox} = 0.5 μm and 1 μm produce a similar number of interference fringes, while the case with D_{ox} = 10 μm produces significantly more fringes.

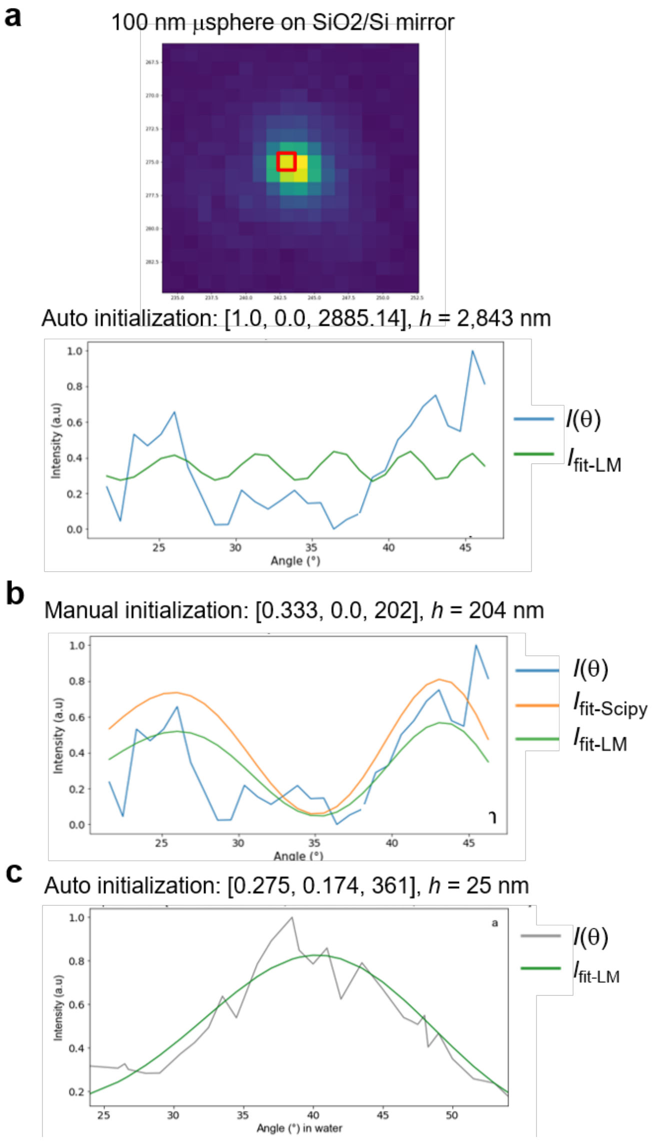

Supplementary Fig. 8. **a**. The blue line represents noisy raw fluorescence intensity data (λ = 488 nm excitation) from the pixel located in the red box at the top fluorescence image of a 100 nm (in diameter) microsphere positioned on the SiO_2/Si mirror. The original Levenberg-Marquardt least square fitting algorithm was applied to fit the curve, resulting in a poor fit (Ifit-LM). **b**. A different initialization parameter set (*a*, *b*, *h*; see Supplementary Fig. 6) was used to fit the raw data using the Scipy.optimize.least_squares function in Python and the Levenberg-Marquardt least square fitting algorithm. This yielded better fits than in (a), but the fitted height value, 204 nm, significantly deviates from the expected 100 nm. **c**. A less noisy fluorescence intensity curve was obtained from another microsphere. The fitting in this case was therefore significantly improved compared to the previous two cases (a and b). However, the fitted height value, 25 nm, also significantly deviates from the expected 100 nm.

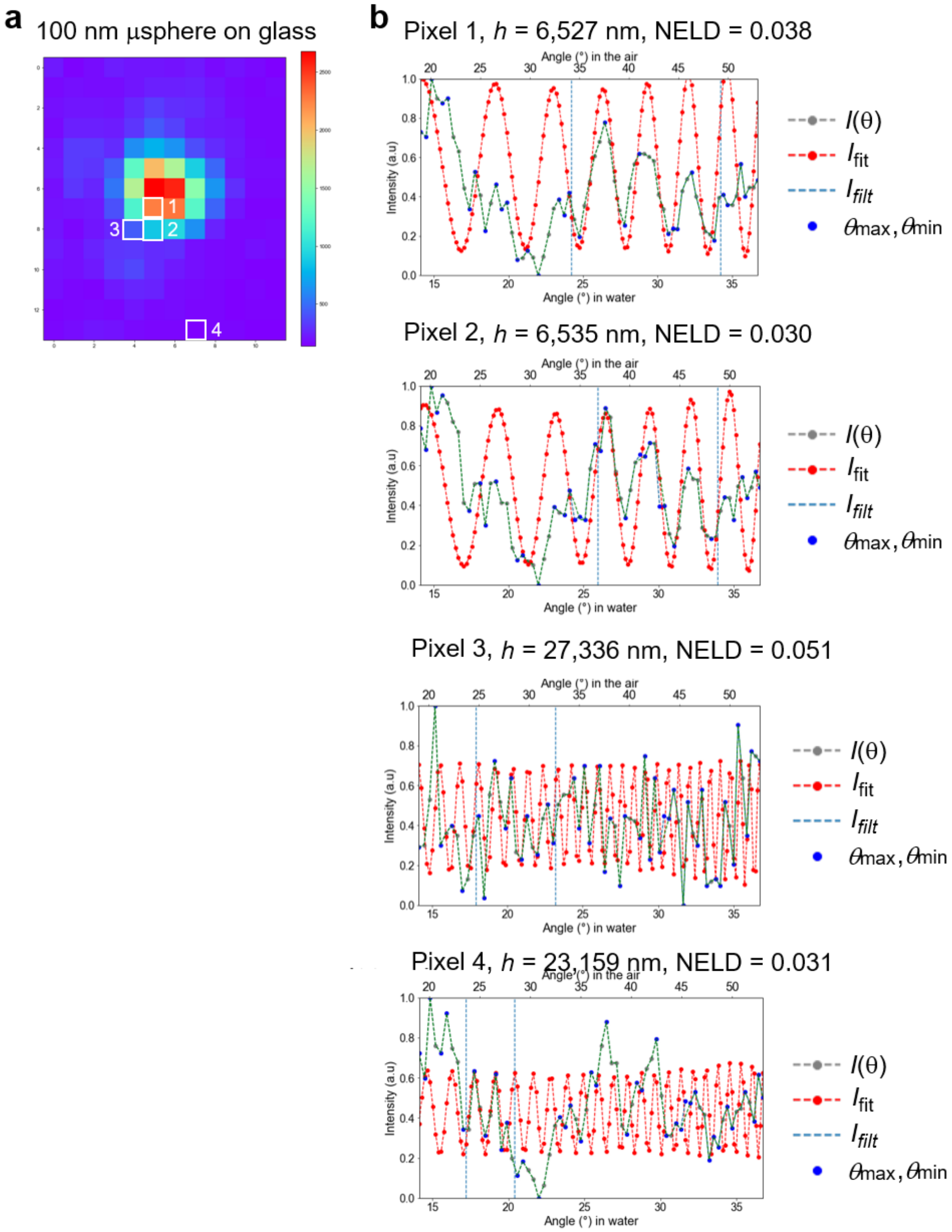

Supplementary Fig. 9. **a**. The fluorescence image displays a microsphere positioned on the bottom glass, approximately 7 µm away from the SiO_2/Si mirror. **b**. Raw fluorescence intensity curves (488 nm excitation) were obtained from four pixel points. Pixels 1 and 2 mainly contain the signal from the microsphere, while pixels 3 and 4 primarily consist of background noise. Our height reconstruction algorithm (see Supplementary Fig. 6) produced excellent fits for all cases, as indicated by the outstanding NELD (< 0.1) values. This was achieved by determining the best initial height parameter and identifying the optimal angle ranges for fitting (between two blue bars).

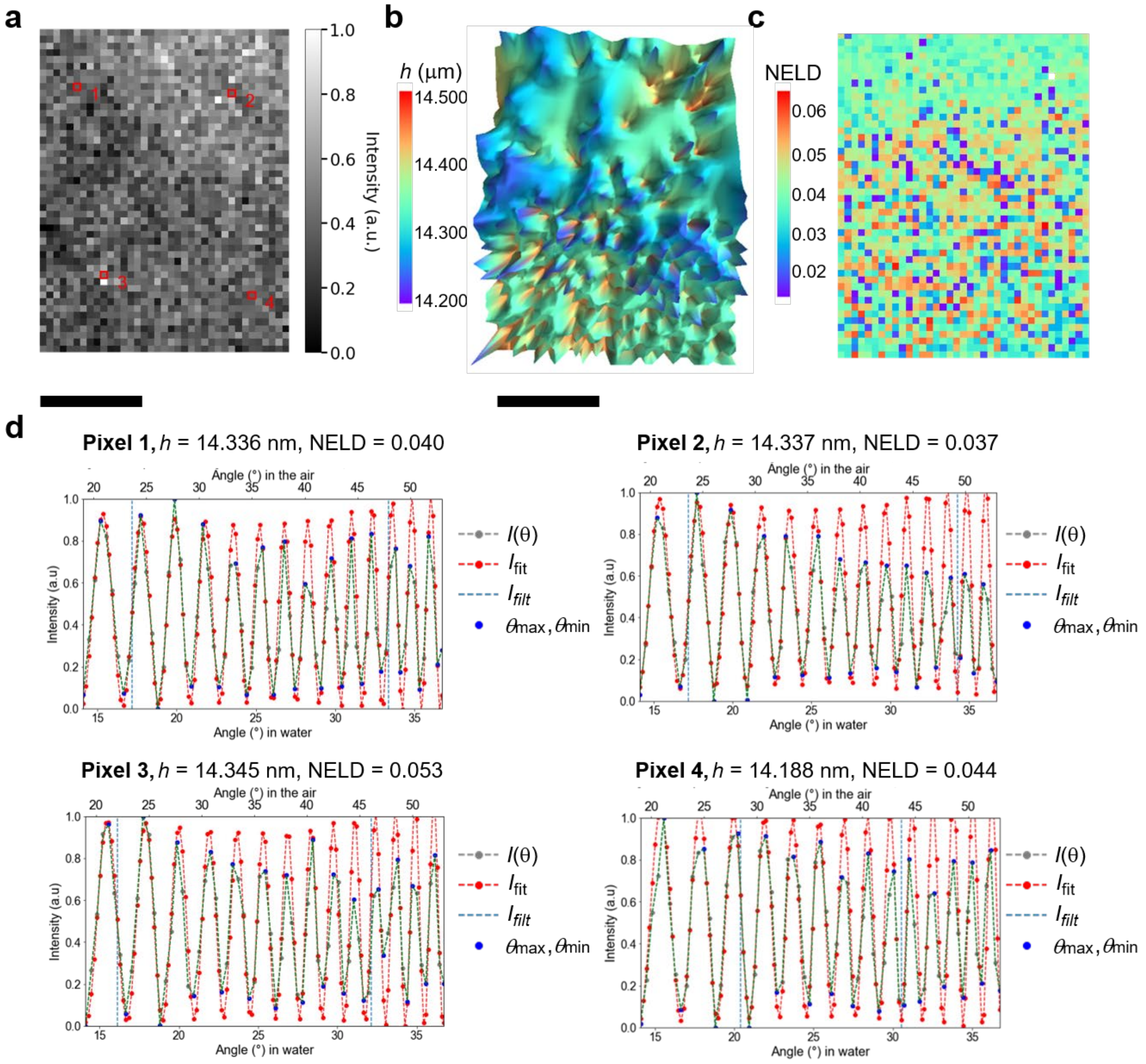

Supplementary Fig. 10. **a**. Raw MAxSIM image of Alexa-488-IgG1 spin-coated on a glass substrate, taken at an incident angle of 14.11° of the 488-nm laser using the 13.7-μm high height-controlled mirror. Four random pixel locations are indicated for demonstrating the high fitting fidelity show in (**d**). Scale bars for **a** and **b** = 2 μm. **b**. The height reconstructed MAxSIM image is represented in 3D. Different heights can be inferred from different color scales. **c.** 2D representation of NELD values, demonstrating excellent fitting fidelity based on the <0.1 NELD values calculated for the selected angle ranges (blue dotted vertical bars shown in **d**) for fitting. **d.** Gray, green, and red dashed lines are raw data, processed raw data, and fitted plots for the four pixels in (a). Blue dots indicate the identified extrema locations. The *h* values were retrieved at 14,336 nm (pixel #1), 14,337 nm (#2), 14,345 nm (#3), and 14.188 nm (#4).

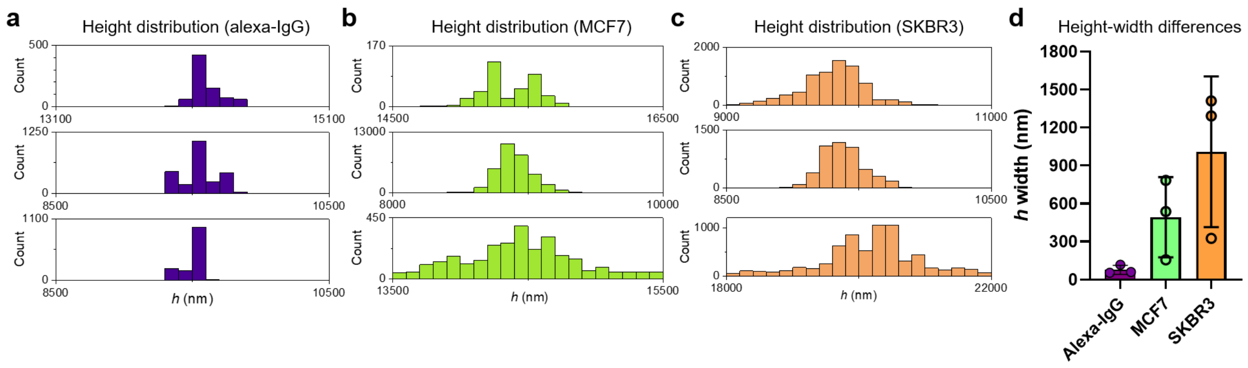

Supplementary Fig. 11. Height distribution plots for the fitted heights with NELD < 0.1 are shown for independent MAxSIM imaging of Alexa 488-IgG1 conjugates spin-coated on a glass surface (**a**; represented by eggplant bar graphs), and WGA-Alexa 555-stained MCF7 (**b**; represented by light green bar graphs) and SKBR3 (**c**; represented by orange bar graphs) cells. **d.** The individual Gaussian width values for the height distributions (a-c), and the mean (represented by the upper line of the box plot) and standard deviation values (error bars) are plotted, showing a significantly narrow height distribution (width: 77 ± 35 nm) for Alexa 488-IgG1, compared to MCF7 (width: 493 ± 315 nm) and SKBR3 (width: 1009 ± 594 nm). Supplementary Data 2 contains the raw data from three separate and independent measurements (n = 3) for the group. Consistent with predictions, the SKBR3 cells on glass exhibited the broadest membrane height distribution, while the distribution was narrowest for Alexa-IgG coated glass.

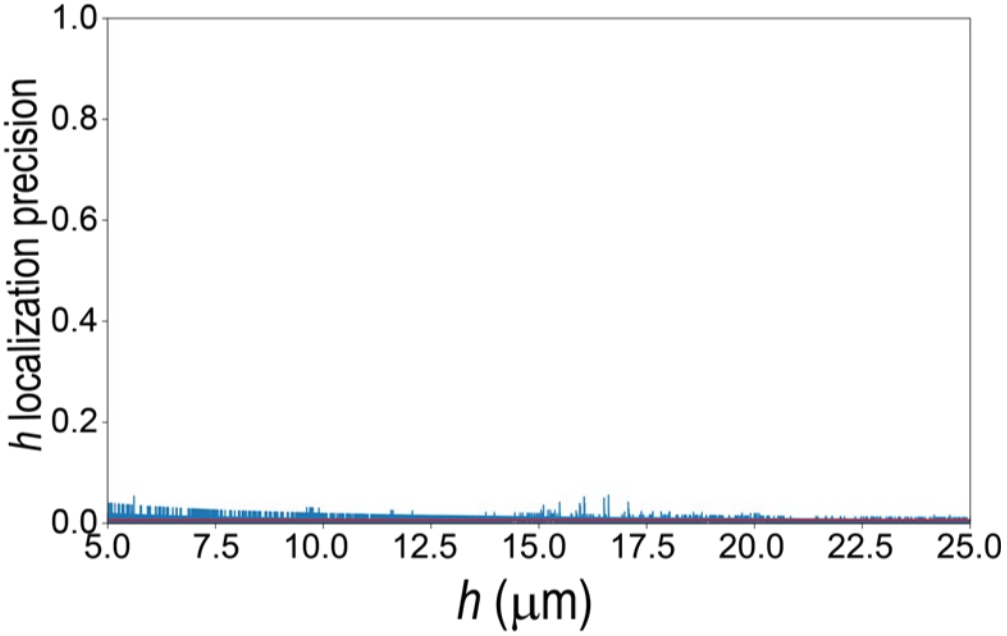

Supplementary Fig. 12. Theoretical height (*h*) localization accuracy (blue line plot) calculated and plotted for *h* = 5–25 μm, every 1 nm. *h* localization accuracy is calculated from the normalized difference of observed (h_o) and theoretical (h_e) heights. Y axis: $|(h_o - h_e)|/h_e$; X axis: h_e = 5–25 μm, every 1 nm. The average localization accuracy is ~0.7% (purple line).

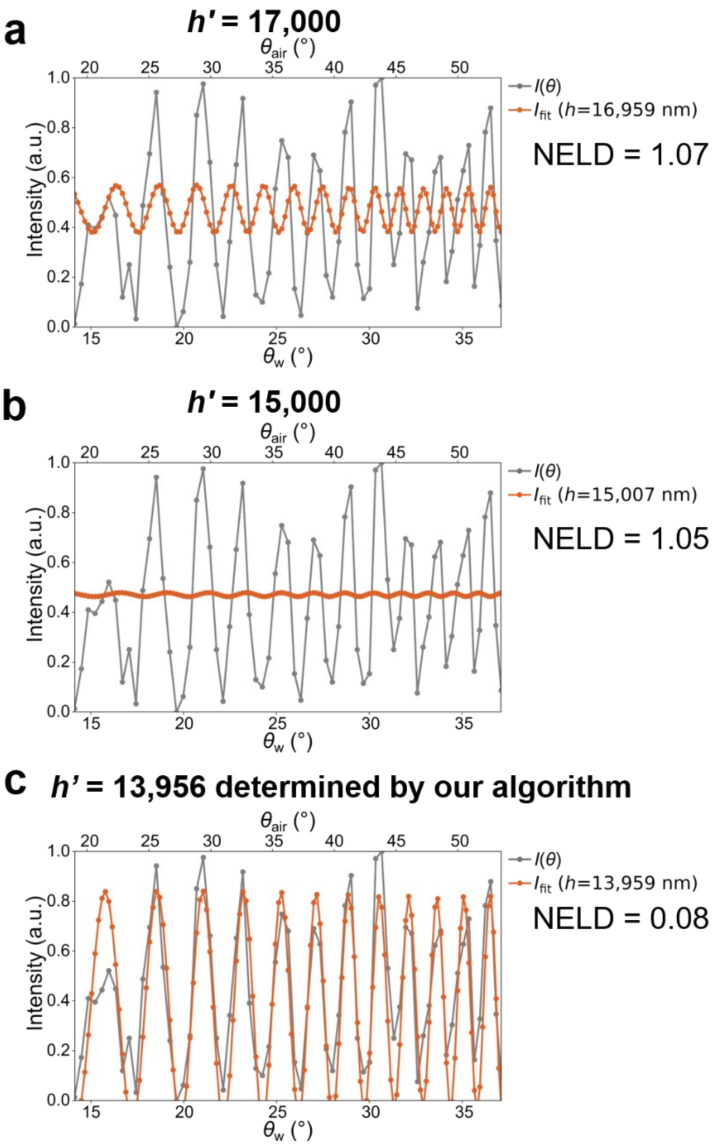

Supplementary Fig. 13. To compare the existing fitting method (a,b), which only applies the least square fitting without determining the appropriate initial height parameter and selecting the optimal angle range for fitting, to our height reconstruction algorithm (c), we used random initial height values (*h*' = 17,000 nm and 15,000 nm for a and b). The existing method leads to poor fit (NELD > 1), while our height reconstruction algorithm finds *h*' = 13,959 nm that is close to the *h* value and shows outstanding fitting fidelity (NELD < 0.1).

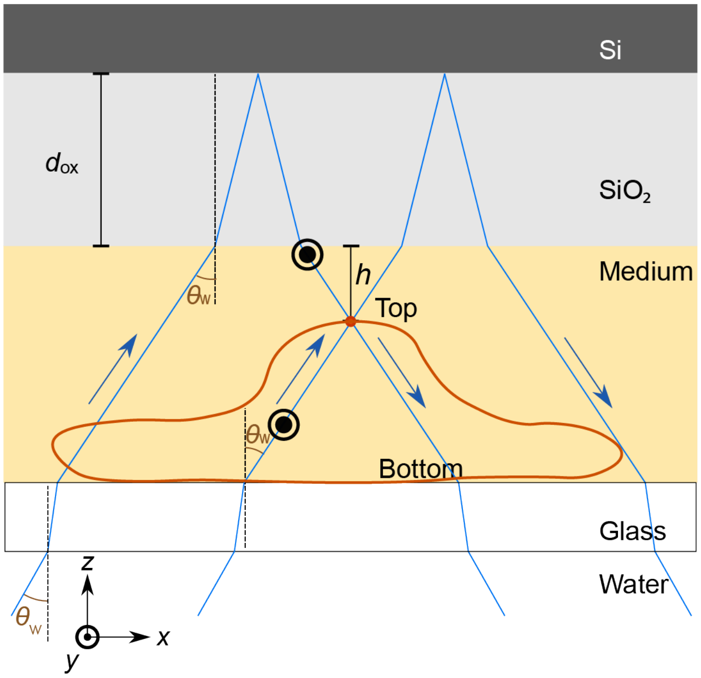

Supplementary Fig. 14. Ray diagram for one-beam MAxSIM geometry for the chromophore location (h from the SiO_2 layer) on a cell placed on the glass substrate. The s-polarized incoming light beams (upper right directions) enter from the water immersion/glass to cell medium at the incidence angle θ_w and reflect off at the Si layer (lower right directions), forming axial interference patterns. d_{ox} is the thickness of the SiO_2 layer.

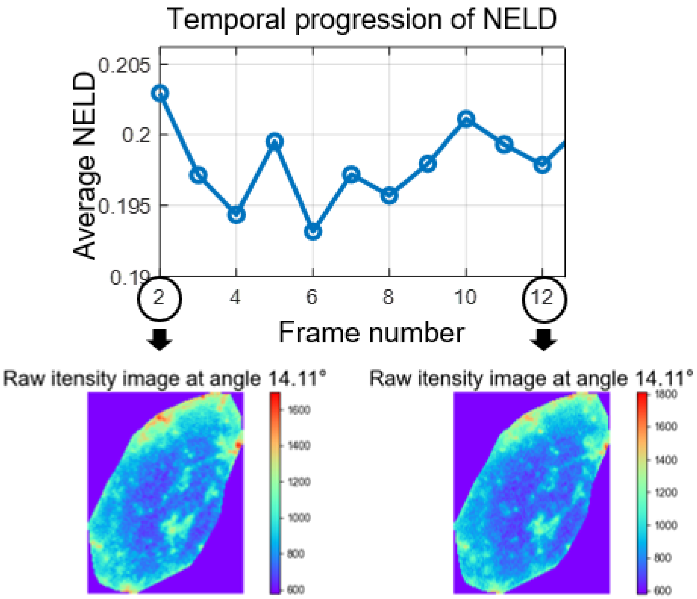

Supplementary Fig. 15. Up: This plot illustrates the temporal progression of NELD values for 11 images of live, WGA-555-stained SKBR3 cells captured at a 50 ms exposure time with a 1.9 s interval, as presented in Supplementary Video 1. The NELD values predominantly stay below, and occasionally just above, 0.2 during this period. This is further evidenced by the raw fluorescent images (shown at the bottom) taken at θ = 14.11º, which display minimal photobleaching.

Techniques	Resolution
3D-STORM	xy ~ 10-30 nm<br>z ~ 30-50 nm
3D-PALM	xy ~ 20-30 nm<br>z ~ 20 - 100 nm
3D-STED	xy ~ 10–50 nm<br>z ~ 50 – 100
iPALM	xy ~ 20 nm<br>z < 50 nm<br>* Imaging depth is limited to 250 nm for λ=600 nm
SAIM	xy: ~ 300 nm (diffraction limited)<br>Z localization accuracy: 5-10 nm
MAxSIM	xy > 120 nm (2D-SIM can be applied)<br>Z localization accuracy : 0.7% of the chromophore's axial position from the Si<br>(e.g., 7 nm axial precision if a chromophore is 1 μm away from the Si)
3D-SIM	xy ~ 100 nm<br>z > 300 nm

Supplementary Table. 1. Comparison of localization accuracy and spatial resolution of various 3D super-resolution imaging and 3D localization techniques, including MAxSIM's theoretical axial localization accuracy.

Supplementary Video 1. Height fluctuation movie of SKBR3 cell plasma membrane stained with WGA-alexa555 conjugates. All height values from reconstruction are retained regardless of the NELD values.

Supplementary Video 2. Height fluctuation movie of SKBR3 cell plasma membrane stained with WGA-alexa555 in conjunction with 3D single molecule tracking of HER2 using αHER2 fab'-QD605 conjugates as probes. All height values from reconstruction are retained regardless of the NELD values.

Supplementary Video 3. Height fluctuation movie of SKBR3 cell plasma membrane stained with WGA-alexa555. All height values from reconstruction are retained regardless of the NELD values.

Supplementary References

1 Paszek, M. J. *et al.* Scanning angle interference microscopy reveals cell dynamics at the nanoscale. *Nature Methods* **9**, 825-+, doi:10.1038/Nmeth.2077 (2012).

2 Born, M. *et al*. Principles of Optics: Electromagnetic Theory of Propagation, Interference and Diffraction of Light, 7th edn. Cambridge University Press, Cambridge, UK doi:10.1017/CBO9781139644181 (1999).

3 Lambacher, A. & Fromherz, P. Fluorescence interference-contrast microscopy on oxidized silicon using a monomolecular dye layer. *Appl Phys a-Mater* **63**, 207-216, doi:Doi 10.1007/Bf01567871 (1996).

4 https://docs.scipy.org/doc/scipy-1.8.0/html-scipyorg/reference/generated/scipy.signal.find_peaks.html.
