# Supplementary material for: MAxSIM: multi-angle-crossing structured illumination microscopy with height-controlled mirror for 3D topological mapping of live cells
Source: Commun Biol. 2023 Oct 12;6:1034. doi: 10.1038/s42003-023-05380-2 (PMC10570291; doi:10.1038/s42003-023-05380-2)
Supplement: Supplementary file 3 — Description of Additional Supplementary Data [file 42003_2023_5380_MOESM3_ESM.docx]

**Description of Additional Supplementary Files**

**File name:** Supplementary Data 1

**Description:** The source data behind the graph in Supplementary Figure 3.

**File name:** Supplementary Data 2

**Description:** The source data behind the graph in Supplementary Figure 11.
